# A GalNAc/Gal-specific lectin from the sea mussel *Crenomytilus grayanus* modulates immune response in macrophages and in mice

**DOI:** 10.1038/s41598-017-06647-5

**Published:** 2017-07-24

**Authors:** Oleg V. Chernikov, Wei-Ting Wong, Lan-Hui Li, Irina V. Chikalovets, Valentina I. Molchanova, Shih-Hsiung Wu, Jiahn-Haur Liao, Kuo-Feng Hua

**Affiliations:** 1 G.B. Elyakov Pacific Institute of Bioorganic Chemistry FEB RAS, Vladivostok, Russia; 2 Department of Biotechnology and Animal Science, National Ilan University, Ilan, Taiwan; 3 Graduate Institute of Life Sciences, National Defense Medical Center, Taipei, Taiwan; 4 Department of Laboratory Medicine, Lisen, Chinese Medicine and Kunming Branch, Taipei City Hospital, Taipei, Taiwan; 5 School of Natural Sciences, Far Eastern Federal University, Vladivostok, Russia; 6 Institute of Biological Chemistry, Academia Sinica, Taipei, Taiwan; 7 Department of Pathology, Tri-Service General Hospital, National Defense Medical Center, Taipei, Taiwan

## Abstract

A GalNAc/Gal-specific lectin (CGL) from the edible mussel *Crenomytilus grayanus* has been demonstrated to exhibit antibacterial properties. However, the mechanism of immune modulation by CGL in mammalian cells remains unclear. Here, we demonstrated that CGL can activate immune responses in macrophages and in mice. In the *in vitro* cell models, CGL induced tumour necrosis factor-α and interleukin-6 secretion in mouse RAW264.7 macrophages, mouse bone marrow-derived macrophages, human THP-1 macrophages, human peripheral blood mononuclear cells and human blood monocyte-derived macrophages. The CGL-mediated cytokine production was regulated by reactive oxygen species, mitogen-activated protein kinases, protein kinase C-α/δ and NF-κB. Interestingly, in lipopolysaccharide-activated macrophages, CGL induced endotoxin tolerance (characterized by the downregulation of nitric oxide, inducible nitric oxide synthase, interleukin-6 and cyclooxygenase II) via the downregulation of IRAK2 expression, JNK1/2 phosphorylation and NF-κB activation. CGL also slightly increased the bactericidal activity of macrophages and induced cytokine production in mouse models. Overall, our data indicate that CGL has the potential to be used as an immune modulator in mammals.

## Introduction

Innate immunity is the first line of the immune system that responds to invasive pathogens and activates adaptive immunity to defend the host from infection by other organisms. One of the first responses of innate immunity is inflammation, which is characterized by the production of inflammatory cytokines^1^. Macrophages play an important role in host defence by ingesting pathogens and activating adaptive immunity through antigen presentation and cytokine production^2^. However, the over-production of cytokines by activated macrophages has been shown to be harmful to health^3^.

Lectins are carbohydrate-binding proteins found in bacteria, viruses, yeast, plants and animals. They serve various biological functions and have antimicrobial, anti-cancerous^4, 5^ and immune regulatory properties in mammals^6^. The phylum Mollusca is one of the largest and most important groups in the animal kingdom. Because molluscs live in very exigent, competitive and aggressive surroundings, a number of different types of substances have been procured from the animals. Various terrestrial environmental factors produce specific and potent active compounds. Among the produced lectins of Mollusca representatives, those isolated from species having economic or medical value, such as mussels or oysters, are of high interest. These lectins can be used in minimally invasive therapies. A significant number of lectins isolated from bivalves cannot be categorized into any existing classes of lectins^7^. Interestingly, the diversity of marine organisms provides new origins and sources of lectins with unusual properties^8^. For example, lectins from the brown alga *Hizikia fusiformis* have free radical scavenging activity^9^. Additionally, a C-type lectin from the oyster *Crassostrea gigas* binds to and enhances phagocytic activity against the bacterium, *Vibrio splendidus* ^10^. We have previously identified a new GalNAc/Gal-specific lectin from the mussel, *Mytilus trossulus*, showing antimicrobial and antifungal activities^11^. Several other lectins have been discovered from bivalves, including C-type lectins, galectins, fibrinogen and C1q-binding lectins and F-type lectins (i.e., ficolins)^12–16^. These findings confirm that bivalve molluscs, including mussels, may be important in the study of lectins and carbohydrate-dependent processes.

In a prior study, we identified a novel GalNAc/Gal-specific lectin (CGL) from the edible sea mussel *Crenomytilus grayanus* that agglutinates human, mouse, and rabbit erythrocytes^17^. We used cDNA sequencing to determine the amino acid sequence of CGL and found that CGL exhibited antibacterial and antifungal activities in the shellfish by being involved in the recognition and clearance of bacterial pathogens^18, 19^. Recently, we determined the crystal structure of CGL, which revealed the presence of a β-trefoil fold that dimerizes into a dumbbell-shaped quaternary structure in the protein^20^.

In this study, the immune modulation properties of CGL in mammals were investigated. We demonstrated that CGL acts as a potentially useful immune modulatory reagent by producing cytokines in macrophages and in mice, increasing the bactericidal activity of macrophages and inducing endotoxin tolerance in macrophages.

## Results

### CGL activates macrophages in an LPS-independent manner

**Figure 1:**
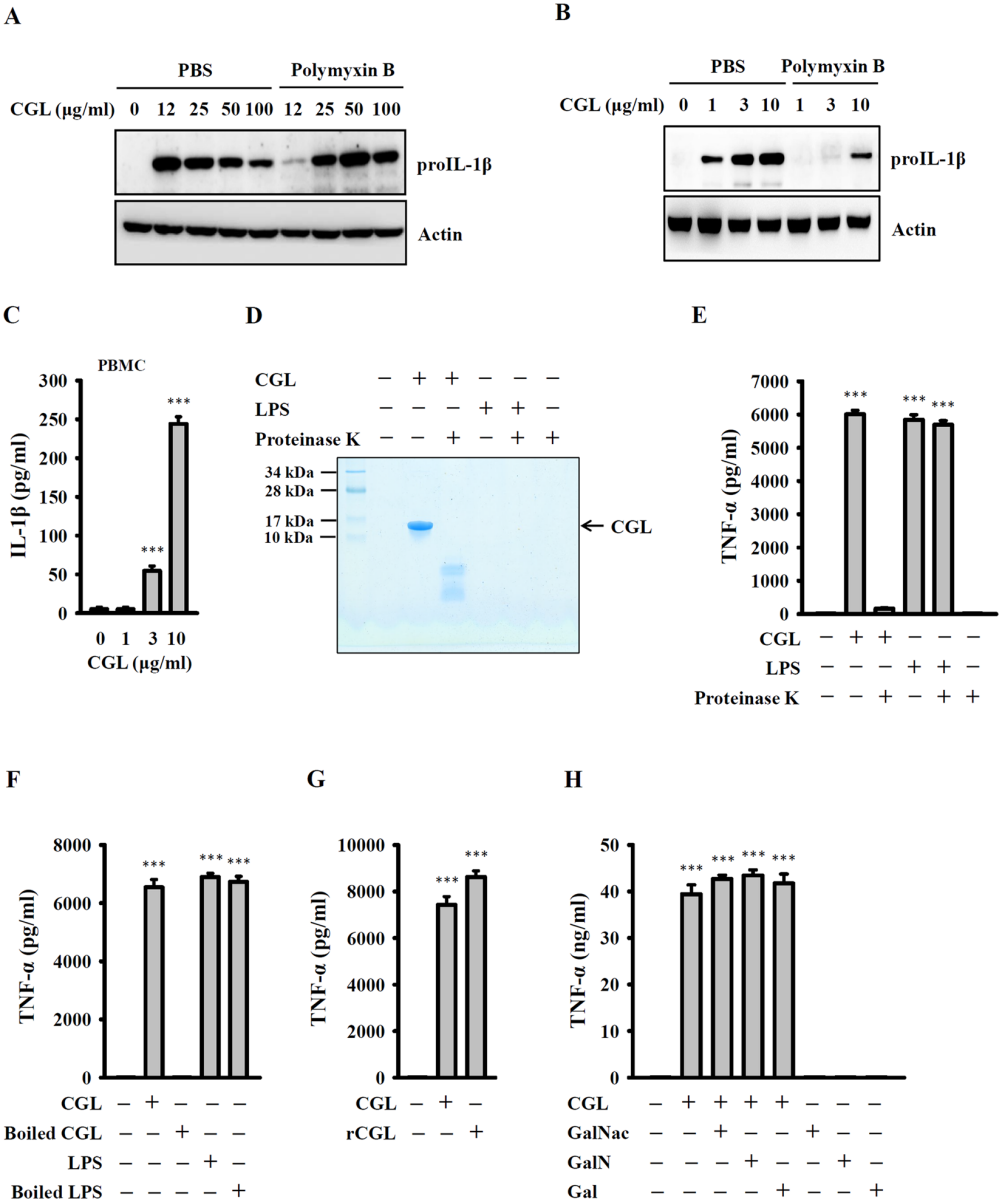
CGL activated macrophages in an LPS independent manner. (**A**,**B**) J774A.1 macrophages were incubated for 30 min with or without polymyxin B (PMB, 10 µg/ml) followed by 6 h incubation with or without CGL. The levels of proIL-1β in the cell lysates were measured by Western blot. (**C**) PBMCs were incubated for 24 h with or without CGL. The levels of IL-1β in the culture medium were measured by ELISA. (**D**) CGL or LPS was incubated with or without proteinase K for 3 h at 50 °C, and then the samples were analysed by SDS-PAGE and Coomassie Blue staining. (**E**) J774A.1 macrophages were incubated for 24 h with or without untreated or proteinase K-treated CGL (10 μg/ml) or LPS (1 μg/ml). (**F**) J774A.1 macrophages were incubated for 24 h with or without untreated or boiled CGL (10 μg/ml) or LPS (1 μg/ml). (**G**) J774A.1 macrophages were incubated for 24 h with or without natural isolated CGL (10 μg/ml) or recombinant CGL (rCGL) (10 μg/ml). (**H**) RAW264.7 macrophages were incubated for 24 h with or without CGL or monosaccharide-incubated CGL. The levels of TNF-α in the culture medium of (**E**–**H**) were measured by ELISA. The ELISA data are expressed as the means ± SD of three separate experiments. * and *** indicate significant differences at the levels of *p* < 0.05 and *p* < 0.001, respectively, compared to control cells. The blots in (**A**) and (**B**) were cropped; full-length blots are included in the “Supplementary Information”.

To test whether CGL activated macrophages, we detected the expression levels of IL-1β precursor (proIL-1β) in CGL-stimulated macrophages. As shown in Fig. 1A, cells treated with 12–100 μg/ml of CGL expressed proIL-1β, although not in a dose-dependent manner. To rule out the possibility of LPS contamination in CGL, we tested the effects of CGL on the induction of proIL-1β in macrophages in the presence and absence of polymyxin B (PMB), a positively charged LPS-sequestering agent. As shown in Fig. 1A, CGL-induced proIL-1β expression was not reduced by PMB at CGL concentrations of 25, 50 and 100 μg/ml. However, PMB significantly inhibited CGL-induced proIL-1β expression at 12 μg/ml CGL. To further rule out the possibility of LPS contamination in CGL, we measured the LPS-content in CGL by the limulus amoebocyte lysate test. The data showed that CGL contained less than 0.1 EU of LPS/1 mg. We further investigated the dose response of CGL (ranging from 1–10 μg/ml) on proIL-1β expression. We found that low concentrations of CGL could induce proIL-1β expression in a dose-dependent manner, and these effects were reduced, but not completely inhibited by PMB (Fig. 1B). However, in cell culture medium, the pI of CGL was acidic (6.12), making it negatively charged. This could have resulted in the reduced proIL-1β expression in the presence of PMB. CGL induced proIL-1β expression and IL-1β secretion in peripheral blood mononuclear cells (Fig. 1C).

Due to concerns about possible LPS contamination in our tested samples and to demonstrate that the effects observed in this study were due to CGL and not LPS, we performed additional experiments. Based on the chemical properties of CGL and LPS, only CGL can be digested by proteinase K. CGL and LPS were incubated with or without proteinase K for 2 h, and the samples were evaluated by SDS-PAGE and Coomassie Blue staining. We found that CGL was completely digested after proteinase K treatment (Fig. 1D). Importantly, CGL completely lost TNF-α induction activity after proteinase K treatment, but proteinase K-treated LPS significantly induced TNF-α production (Fig. 1E). Then, we destroyed CGL by boiling the samples for 10 min. We found that CGL lost TNF-α induction activity after boiling, but boiled LPS still significantly induced TNF-α production (Fig. 1F). Finally, we expressed recombinant CGL and removed possible LPS contamination by purifying recombinant CGL using an endotoxin removal resin. We found that the recombinant CGL showed similar TNF-α induction activity compared with naturally isolated CGL (Fig. 1G). These results indicated that the effects observed in this study were due to CGL but not LPS. In our previous study^19^, CGL binds to N-acetyl-D-galactosamine (GalNAc), galactosamine (GalN) and galactose (Gal). To investigate whether CGL-mediated TNF-α production in macrophages could be inhibited by GalNAc, GalN and Gal, CGL was separately incubated with each monosaccharide at 4 °C for 24 h. We found that the monosaccharides did not affect CGL-mediated TNF-α production, suggesting that the CGL induced cytokine production was independent of its sugar binding property (Fig. 1H).

### CGL induces cytokine production in macrophages

**Figure 2:**
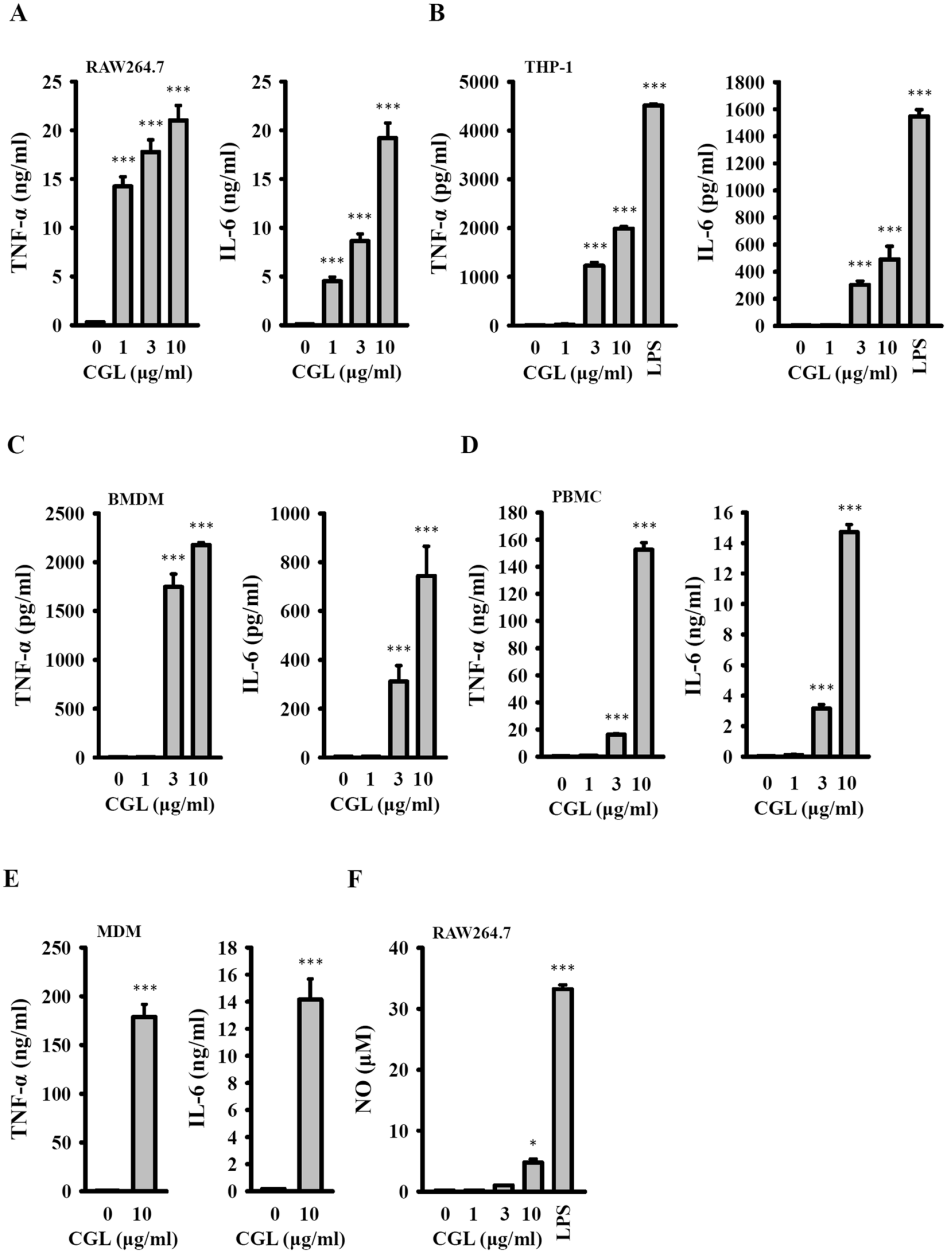
CGL induced cytokine production in macrophages. (**A**) RAW264.7 macrophages, (**B**) human THP-1 macrophages, (**C**) mouse bone marrow-derived macrophages, (**D**) PBMCs and (**E**) MDMs were incubated for 24 h with or without CGL or LPS (1 µg/ml). The levels of TNF-α and IL-6 in the culture medium were measured by ELISA. (**F**) RAW264.7 macrophages were incubated for 24 h with or without CGL or LPS (1 µg/ml). The levels of NO in the culture medium were measured by Griess reaction. The NO and ELISA data are expressed as the means ± SD of three separate experiments. * and *** indicate significant differences at the levels of *p* < 0.05 and *p* < 0.001, respectively, compared to control cells.

To confirm the immune activation properties of CGL, the cytokine induction activity of CGL was investigated. We found that CGL induced TNF-α and IL-6 production in the mouse macrophage cell line RAW264.7 (Fig. 2A) and in the human macrophage cell line THP-1 (Fig. 2B). CGL not only induced cytokine production in macrophages cell lines but also induced cytokine production in primary cells. We demonstrated that CGL induced TNF-α and IL-6 production in mouse bone marrow-derived macrophages (Fig. 2C), human peripheral blood mononuclear cells (Fig. 2D) and human blood monocyte-derived macrophages (Fig. 2E). Additionally, as shown in Fig. 2F, we found that even a CGL concentration of 10 μg/ml was only slightly able to induce NO generation in macrophages (4 μM) compared with 1 μg/ml of LPS, which significantly induced NO generation (33 μM). These results indicated that CGL activated macrophages independently of LPS.

### ROS and PKCα/δ regulate CGL-mediated TNF-α, IL-6 and COX-2 expression

**Figure 3:**
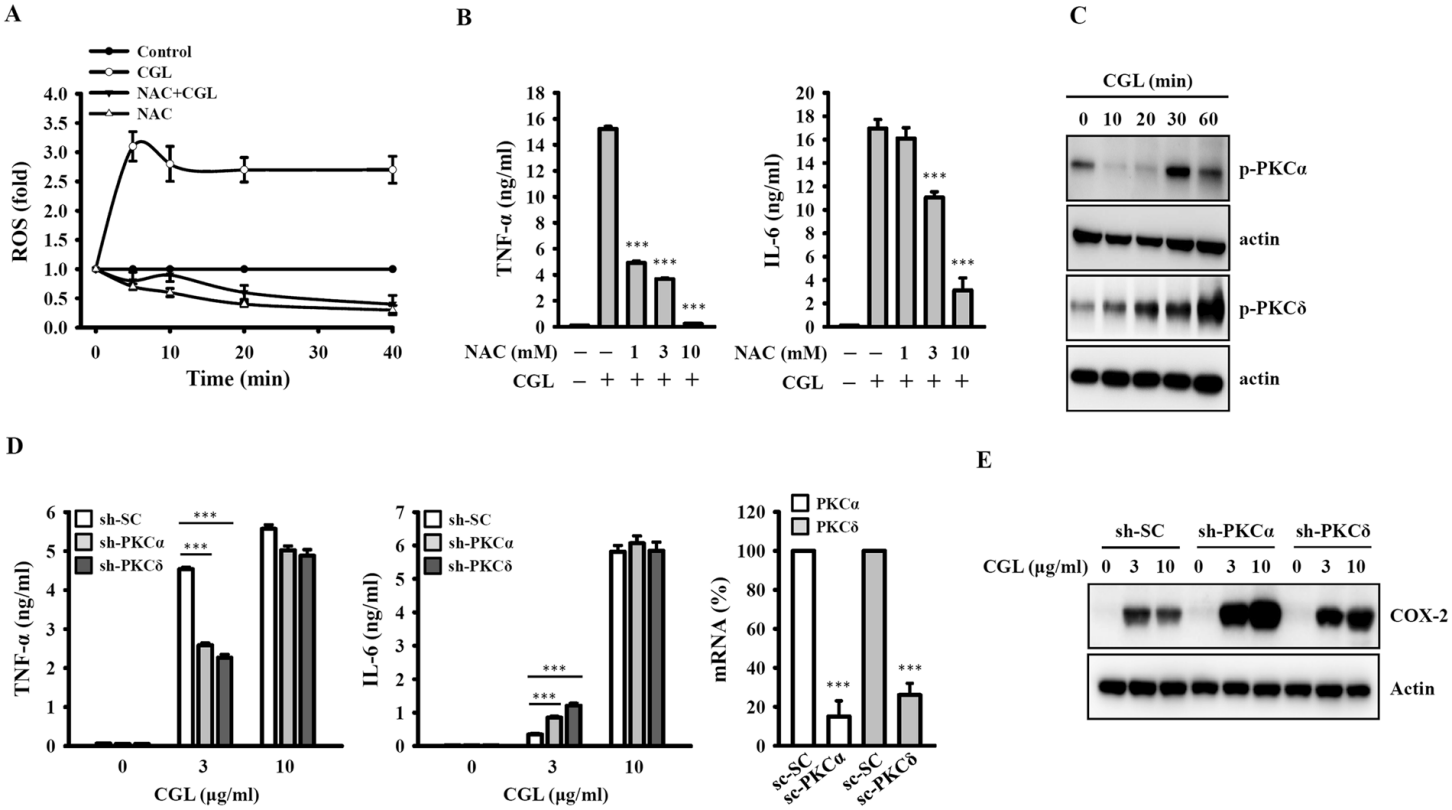
ROS and PKCα/δ regulate CGL-mediated TNF-α, IL-6 and COX-2 expression. (**A**) RAW264.7 macrophages were incubated for 30 min with or without NAC (10 mM) and then for 0–40 min with or without CGL (10 µg/ml). The levels of ROS in the cells were measured by 2′,7′-dichlorofluorescein diacetate. (**B**) RAW264.7 macrophages were incubated for 30 min with or without NAC (10 mM) and then for 24 h with or without CGL. The levels of TNF-α and IL-6 in the culture medium were measured by ELISA. (**C**) RAW264.7 macrophages were incubated for 0–60 min with or without CGL (10 µg/ml). The phosphorylation levels of PKCα and PKCδ were assayed by Western blot. (**D** and **E**) The mRNA expression levels of PKCα and PKCδ were assayed by real-time PCR. The sh-SC, sh-PKCα and sh-PKCδ cells were incubated for 24 h with or without CGL. The levels of TNF-α and IL-6 in the culture medium were measured by ELISA (**D**), and COX-2 in the cell lysates were measured by Western blot (**E**). Western blot results are representative of those obtained in three different experiments. The ELISA data are expressed as the means ± SD of three separate experiments. ***Indicates a significant difference at the level of *p* < 0.001 compared to control cells. The blots in (**C**) and (**E**) were cropped; full-length blots are included in the “Supplementary Information”.

ROS play important roles in cellular signalling and regulate cytokine expression in activated macrophages^21, 22^. CGL induced ROS generation in macrophages; this effect was inhibited by the ROS scavenger N-acetyl-cysteine (NAC) (Fig. 3A). NAC also significantly reduced TNF-α and IL-6 secretion in CGL-activated macrophages (Fig. 3B). These results indicated that CGL mediated TNF-α and IL-6 secretion through ROS associated pathways. PKC is an important signalling molecule controlling the expression of inflammatory mediators in macrophages^21, 23, 24^. We found that the phosphorylation levels of PKCα and PKCδ were increased by CGL treatment (Fig. 3C). To elucidate the role of PKCα and PKCδ in the regulation of TNF-α and IL-6 secretion in CGL-activated macrophages, we used macrophages stably transfected with shRNA plasmids targeting PKCα (sh-PKCα) and PKCδ (sh-PKCδ). We found that TNF-α secretion levels in low dose CGL-activated sh-PKCα and sh-PKCδ cells (3 μg/ml) were significantly lower than in cells stably transfected with a control shRNA plasmid encoding a scrambled shRNA sequence (sh-SC). However, such a difference was not observed under high doses (10 μg/ml) of CGL (Fig. 3D). Conversely, CGL-induced IL-6 secretion levels in sh-PKCα and sh-PKCδ cells were higher than those in sh-SC cells. Again, these effects were only observed in low-dose CGL-activated cells (Fig. 3D). Finally, CGL induced COX-2 expression in sh-SC cells. This effect was enhanced in sh-PKCα and sh-PKCδ cells, with the highest expression in the sh-PKCα cells (Fig. 3E). These results indicated that PKCα and PKCδ may play different roles in CGL-mediated TNF-α, IL-6 and COX-2 expression.

### CGL induces TNF-α and IL-6 secretion through MAPK

**Figure 4:**
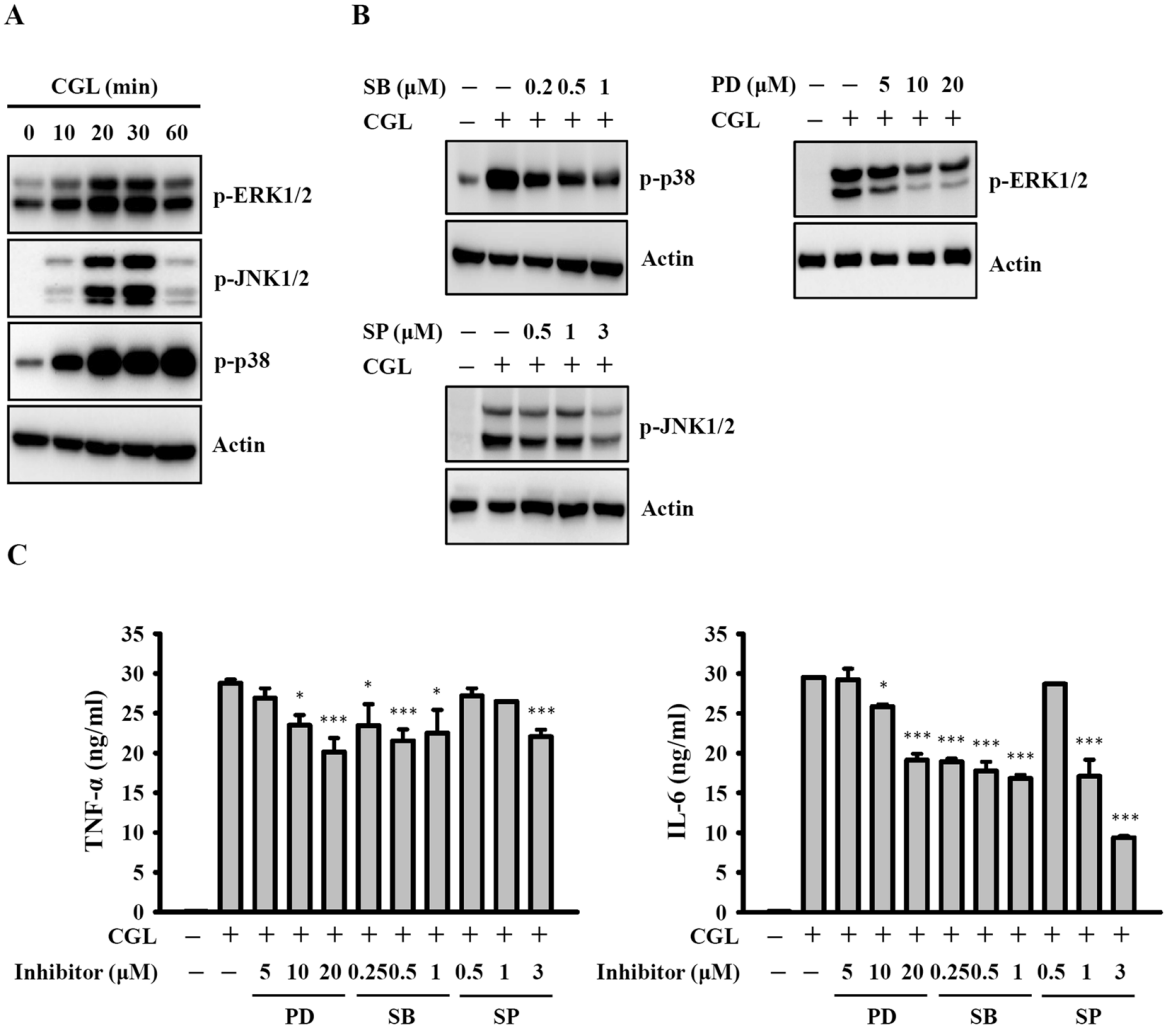
CGL induced TNF-α and IL-6 secretion through MAPK. (**A**) RAW264.7 macrophages were incubated for 0–60 min with or without CGL (10 µg/ml). Phosphorylation levels of ERK1/2, JNK1/2 and p38 were assayed by Western blot. (**B**) RAW264.7 macrophages were incubated for 30 min with or without MAPK inhibitors PD, SB, and SP, followed by 20 min incubation with or without CGL (10 µg/ml). The phosphorylation levels of MAPK in the cells were measured by Western blot. (**C**) RAW264.7 macrophages were incubated for 30 min with or without MAPK inhibitors PD, SB, and SP, followed by 24 h incubation with or without CGL (10 µg/ml). The levels of TNF-α and IL-6 in the culture medium were measured by ELISA. Western blot results are representative of those obtained in three different experiments. The ELISA data are expressed as the means ± SD of three separate experiments. * and *** indicate significant differences at the level of *p* < 0.05 and *p* < 0.001, respectively, compared to CGL-treated cells. The blots in (**A**) and (**B**) were cropped; full-length blots are included in the “Supplementary Information”.

The activation of macrophages results in the downstream induction of mitogen-activated protein kinases (MAPKs, including ERK1/2, JNK1/2 and p38), leading to the production of pro-inflammatory mediators^23, 25^. To examine whether the CGL-induced TNF-α and IL-6 secretions were associated with the MAPK signalling pathways, the phosphorylation levels of ERK1/2, JNK1/2 and p38 in CGL-activated macrophages were measured by Western blot. The results showed that the phosphorylation levels of ERK1/2, JNK1/2 and p38 were increased by CGL (Fig. 4A) and that these effects were reduced by specific inhibitors: PD98059 (MEK1 inhibitor), SP600125 (JNK1/2 inhibitor) and SB203580 (p38 inhibitor), respectively (Fig. 4B). In addition, all inhibitors reduced CGL-induced TNF-α and IL-6 secretion (Fig. 4C). Thus, we concluded that CGL induced TNF-α and IL-6 secretion partially through ERK1/2, JNK1/2 and p38 in CGL-activated macrophages.

### CGL induces TNF-α and IL-6 secretion through NF-κB

**Figure 5:**
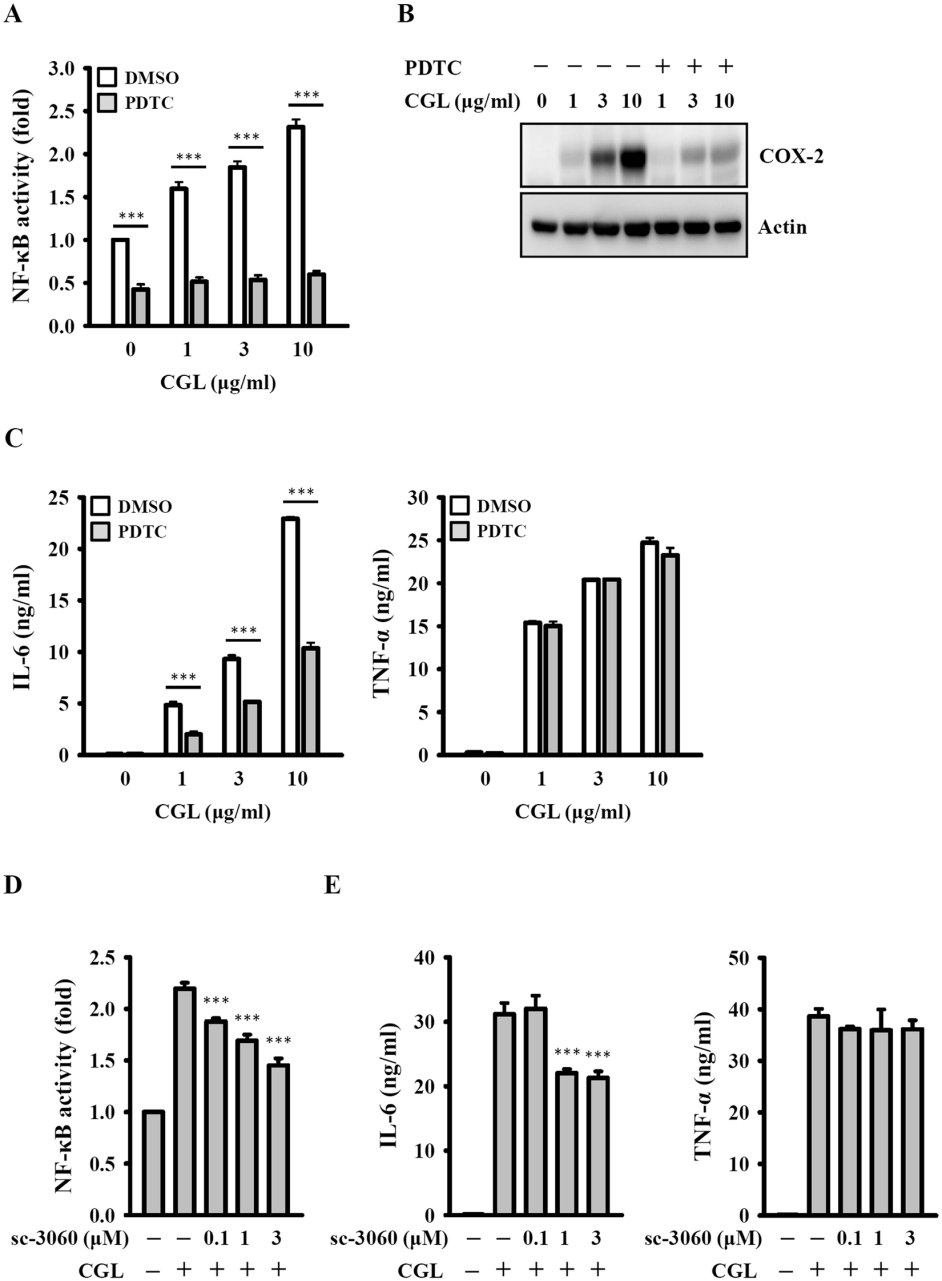
CGL induced TNF-α and IL-6 secretion through NF-κB. (**A**) RAW-Blue^TM^ cells were incubated for 30 min with or without PDTC (30 µM), followed by incubation for 24 h with or without CGL. The activation levels of NF-κB were measured by NF-κB reporter assay. (**B** and **C**) RAW264.7 macrophages were incubated for 30 min with or without PDTC (30 µM), followed by incubation for 24 h with or without CGL. The levels of COX-2 in the cell lysates were measured by Western blot (**B**), and IL-6 and TNF-α in the culture medium were measured by ELISA (**C**). (**D**) RAW-Blue^TM^ cells were incubated for 30 min with or without sc-3060, followed by incubation for 24 h with or without CGL (10 µg/ml). The activation levels of NF-κB were measured by NF-κB reporter assay. (**E**) RAW264.7 macrophages were incubated for 30 min with or without sc-3060, followed by incubation for 24 h with or without CGL (10 µg/ml). The levels of IL-6 and TNF-α in the culture medium were measured by ELISA. Western blot results are representative of those obtained in three different experiments. The ELISA data are expressed as the means ± SD of three separate experiments. ***Indicates a significant difference at the level of *p* < 0.001 compared to CGL-treated cells. The blots in (**B**) were cropped; full-length blots are included in the “Supplementary Information”.

NF-κB is one of the most important transcription factors regulating cytokine expression in macrophages^23, 25^. By assaying NF-κB reporter cells, we found that CGL increased NF-κB transcriptional activity; this was reduced by NF-κB inhibitor PDTC (Fig. 5A). PDTC significantly reduced COX-2 expression (Fig. 5B) and IL-6 secretion (Fig. 5C) in CGL-activated macrophages but did not affect TNF-α secretion (Fig. 5C). The role of NF-κB in CGL-mediated cytokine secretion was further confirmed by using a synthetic cell permeable NF-κB inhibitory peptide. This peptide significantly inhibited CGL-mediated NF-κB activation (Fig. 5D) and IL-6 secretion (Fig. 5E) but did not affect TNF-α secretion (Fig. 5E). These results indicated that the CGL-induced IL-6 secretion and COX-2 expression were partially mediated through NF-κB, whereas the CGL-induced TNF-α secretion was independent of NF-κB.

### ROS act upstream of MAPKs in CGL-activated macrophages

**Figure 6:**
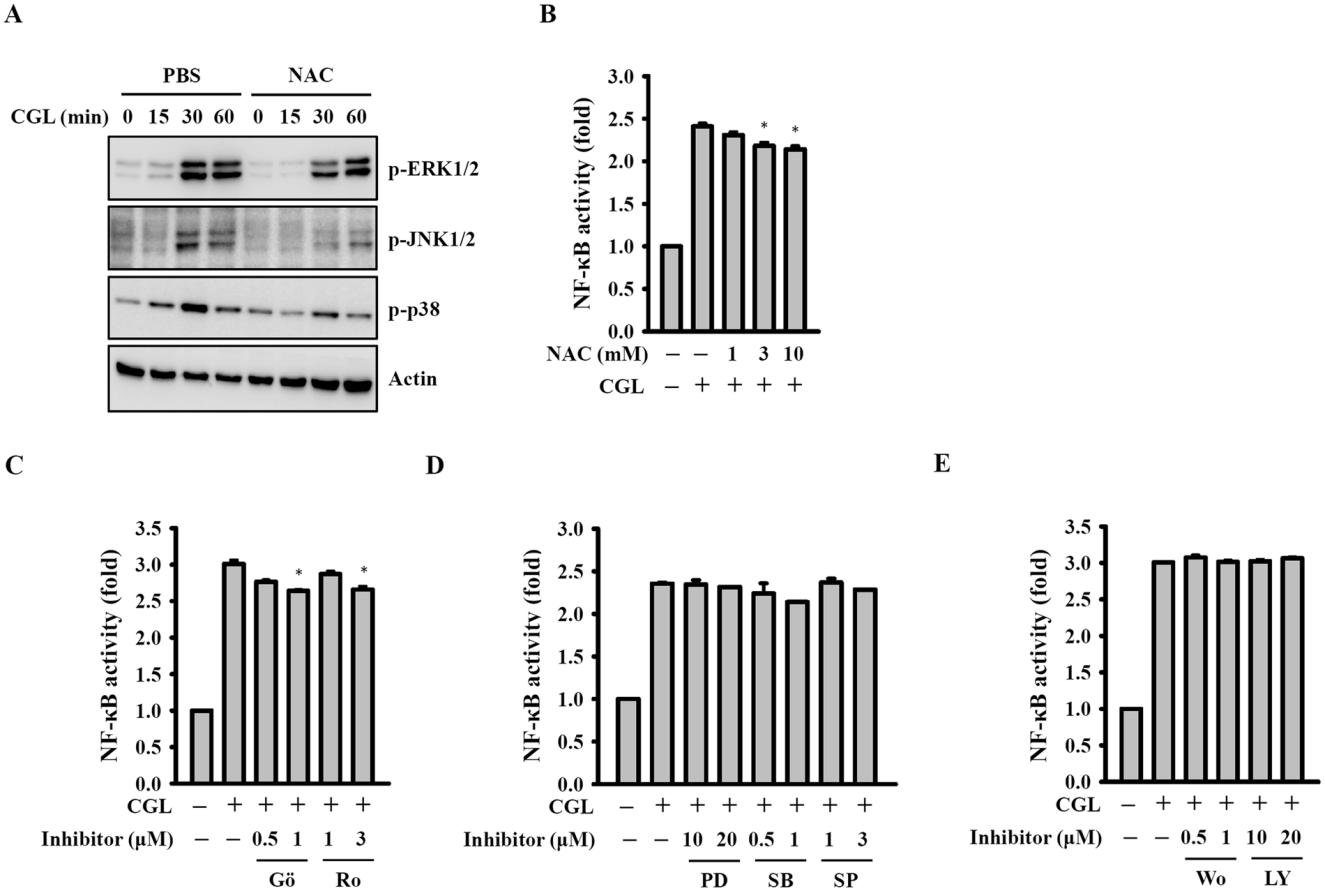
ROS acted upstream of MAPK in CGL-activated macrophages. (**A**) RAW264.7 macrophages were incubated for 30 min with or without NAC (10 mM) and then for 0–60 min with or without CGL (10 µg/ml). The phosphorylation levels of ERK1/2, JNK1/2 and p38 were assayed by Western blot. (**B–E**) RAW-Blue^TM^ cells were incubated for 30 min with or without NAC (**B**), MAPK inhibitor (**C**), PKCα/δ inhibitor (**D**) or PI3-kinase inhibitor (**E**), followed by incubation for 24 h with or without CGL (10 µg/ml). The activation levels of NF-κB were measured by NF-κB reporter assay. Western blot results are representative of those obtained in three different experiments. The NF-κB data is expressed as the means ± SD of three separate experiments. *Indicates a significant difference at the level of *p* < 0.05 compared to CGL-treated cells. The blots in (A) were cropped; full-length blots are included in the “Supplementary Information”.

We tested whether ROS were involved in CGL-induced MAPK activation. We found that CGL-mediated phosphorylation levels of ERK1/2, JNK1/2 and p38 were reduced by the ROS scavenger NAC (Fig. 6A), suggesting that ROS acted upstream of ERK1/2, JNK1/2 and p38 in CGL-activated macrophages. We found that the CGL-induced NF-κB activation was only weakly associated with ROS, as NAC slightly inhibited NF-κB activation (Fig. 6B). Additionally, we determined that CGL-mediated NF-κB activation was also weakly associated with PKCα/δ, as PKCα inhibitor (Gö6970) and PKCδ inhibitor (Rottlerin) slightly reduced NF-κB activation (Fig. 6C). Furthermore, since MAPKs inhibitors, PD98059 (MEK1 inhibitor), SP600125 (JNK1/2 inhibitor), SB203580 (p38 inhibitor), and PI3-kinase inhibitors Wortmannin and LY294002 did not reduce NF-κB activation, we concluded that CGL-mediated NF-κB activation was independent of MAPKs (Fig. 6D) and PI3-kinase (Fig. 6E).

### CGL induces endotoxin tolerance

**Figure 7:**
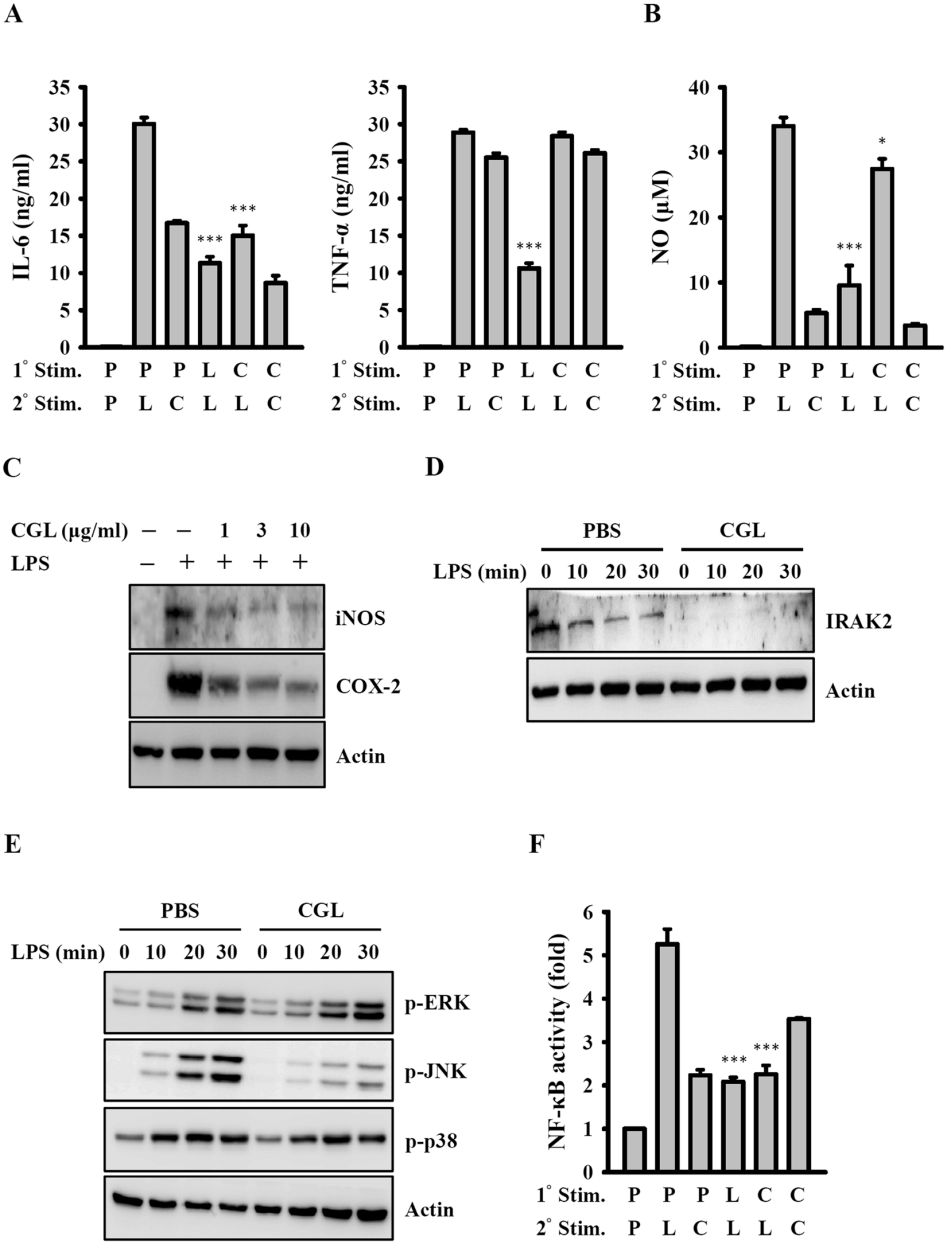
CGL induced endotoxin tolerance. (**A** and **B**) RAW264.7 macrophages were incubated for 24 h with or without CGL (10 µg/ml) or LPS (0.1 µg/ml). After washing, fresh medium was added to the cell cultures followed by a second incubation for 24 h with or without CGL (10 µg/ml) or LPS (1 µg/ml). The levels of IL-6 and TNF-α in the culture medium were measured by ELISA (**A**), the levels of NO in the culture medium were measured by Griess reaction (**B**), and the levels of iNOS and COX-2 in the cell lysates were measured by Western blot (**C**). (**D** and **E**) RAW264.7 macrophages were incubated for 24 h with or without CGL (10 µg/ml) and subsequently for 0–30 min with or without LPS (1 µg/ml). IRAK2 expression (**D**), and phosphorylation levels of ERK1/2, JNK1/2 and p38 (**E**) were assayed by Western blot. (**F**) RAW-Blue^TM^ cells were incubated for 24 h with or without CGL (10 µg/ml) or LPS (0.1 µg/ml). After washing, fresh medium was added to the cell cultures followed by a second incubation for 24 h with or without CGL (10 µg/ml) or LPS (1 µg/ml). The activation levels of NF-κB were measured by NF-κB reporter assay. Western blot results are representative of those obtained in three different experiments. Data for the other assays are expressed as the mean ± SD of three separate experiments. * and *** indicate significant differences at the level of *p* < 0.05 and *p* < 0.001, respectively, compared to LPS-treated cells. P: PBS; C; CGL; L: LPS. The blots in (**A**), (**D**) and (**E**) were cropped; full-length blots are included in the “Supplementary Information”.

The primary exposure of cells to LPS produces reduced responsiveness to a second LPS challenge, a phenomenon known as endotoxin tolerance. Endotoxin tolerance is characterized by a global downregulation of inflammatory gene expression^26^. The induction of endotoxin tolerance augments bacterial clearance and improves survival in mice with sepsis^27^. Although CGL induced an increase in TNF-α and IL-6 secretion in macrophages, the pre-incubation of macrophages with CGL for 24 h produced an endotoxin tolerance-like phenomenon. This also markedly attenuated the response to LPS stimulus by lowering IL-6 secretion (Fig. 7A), NO generation (Fig. 7B), and iNOS and COX-2 expression (Fig. 7C). However, this did not affect TNF-α secretion (Fig. 7A). The endotoxin tolerance-like phenomenon induced by CGL may have resulted from the reduced expression of IRAK2 (Fig. 7D), an important signalling protein that plays a central role in TLR-mediated NF-κB activation pathway^28^. CGL pre-treatment also reduced the phosphorylation levels of JNK1/2 but not ERK1/2 and p38 induction by LPS (Fig. 7E). Additionally, CGL pre-treatment reduced the level of NF-κB activation induced by LPS (Fig. 7F).

### CGL enhances the bactericidal activity of macrophages

**Figure 8:**
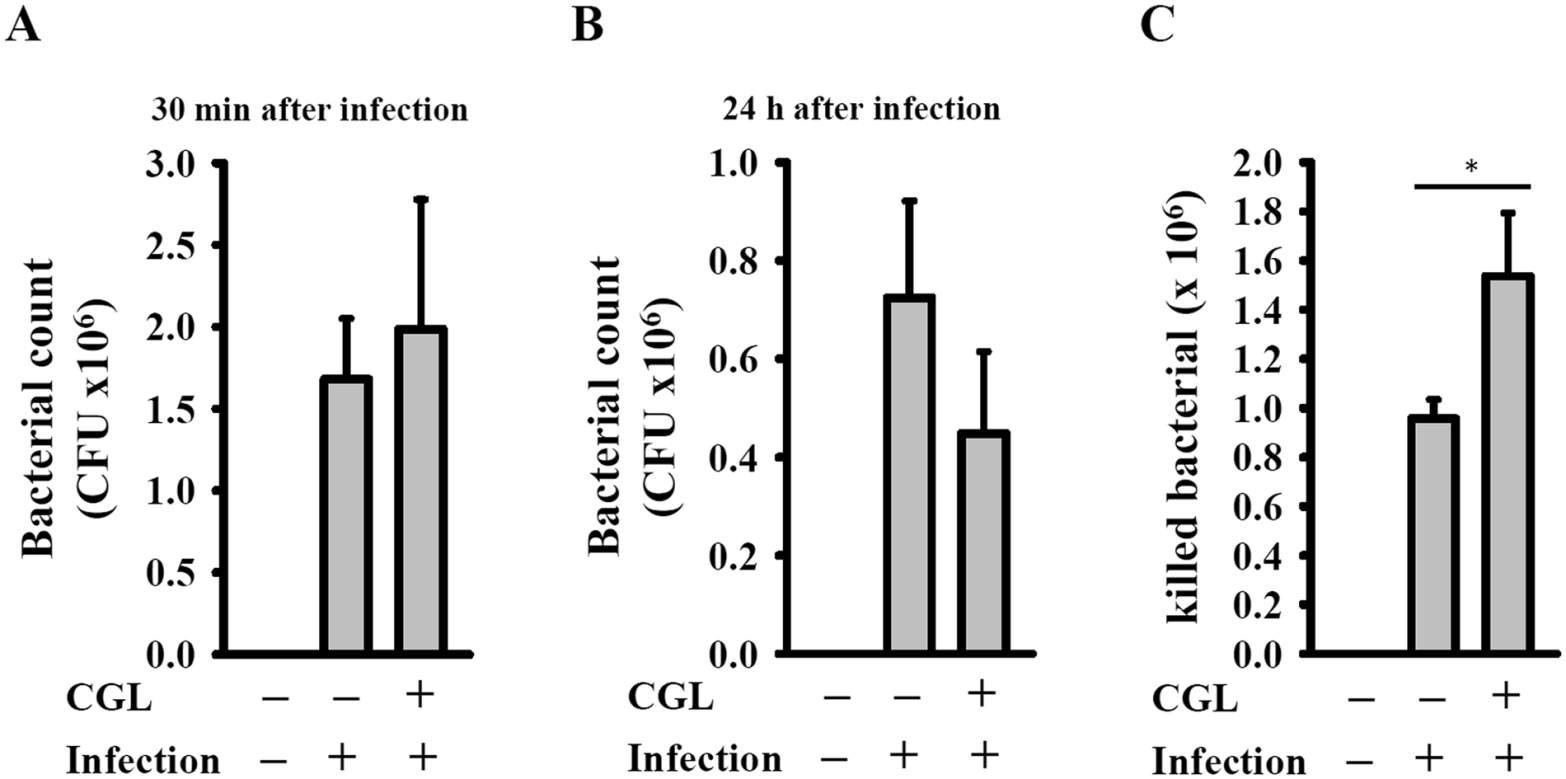
CGL enhanced the bactericidal activity of macrophages. RAW264.7 macrophages were incubated for 24 h with or without CGL (10 µg/ml). After washing, cell cultures were infected with or without *E*. *coli* for 1 h (**A**) and 24 h (**B**), the cells were lysed and the number of engulfed live *E*. *coli* was determined by CFU assay. The number of killed bacteria was calculated by subtracting the 24 h CFU from the 1 h CFU. (**C**). The results are expressed as the means ± SD of three separate experiments. *Indicates a significant difference at the level of *p* < 0.05 compared to the control group without CGL.

The bactericidal activity of macrophages is characterized by increased phagocytosis and killing of bacteria. We used a CFU assay to demonstrate that CGL pre-treatment non-significantly increased the phagocytosis of *E*. *coli* by macrophages (1984 ± 792) compared with control macrophages (1680 ± 370) after 30 min infection (Fig. 8A). These results indicated that CGL pre-treatment slightly increased the phagocytosis of bacteria by macrophages. Nevertheless, these results could have also indicated a reduction in the killing of bacteria by macrophages. Thus, we measured the CFU after 24 h infection and found that the number of CFU in CGL-pre-treated and control cells were 448 ± 166 and 724 ± 196, respectively (Fig. 8B). This indicated that there were approximately 1536 and 956 bacteria (by subtracting the 24 h CFU from the 1 h CFU) killed in the CGL-pre-treated and control cells, respectively, within 24 h (Fig. 8C). Therefore, these results indicated that the incubation of macrophages with CGL slightly increased the bactericidal activity of macrophages.

### CGL induces cytokine expression *in vivo*

**Figure 9:**
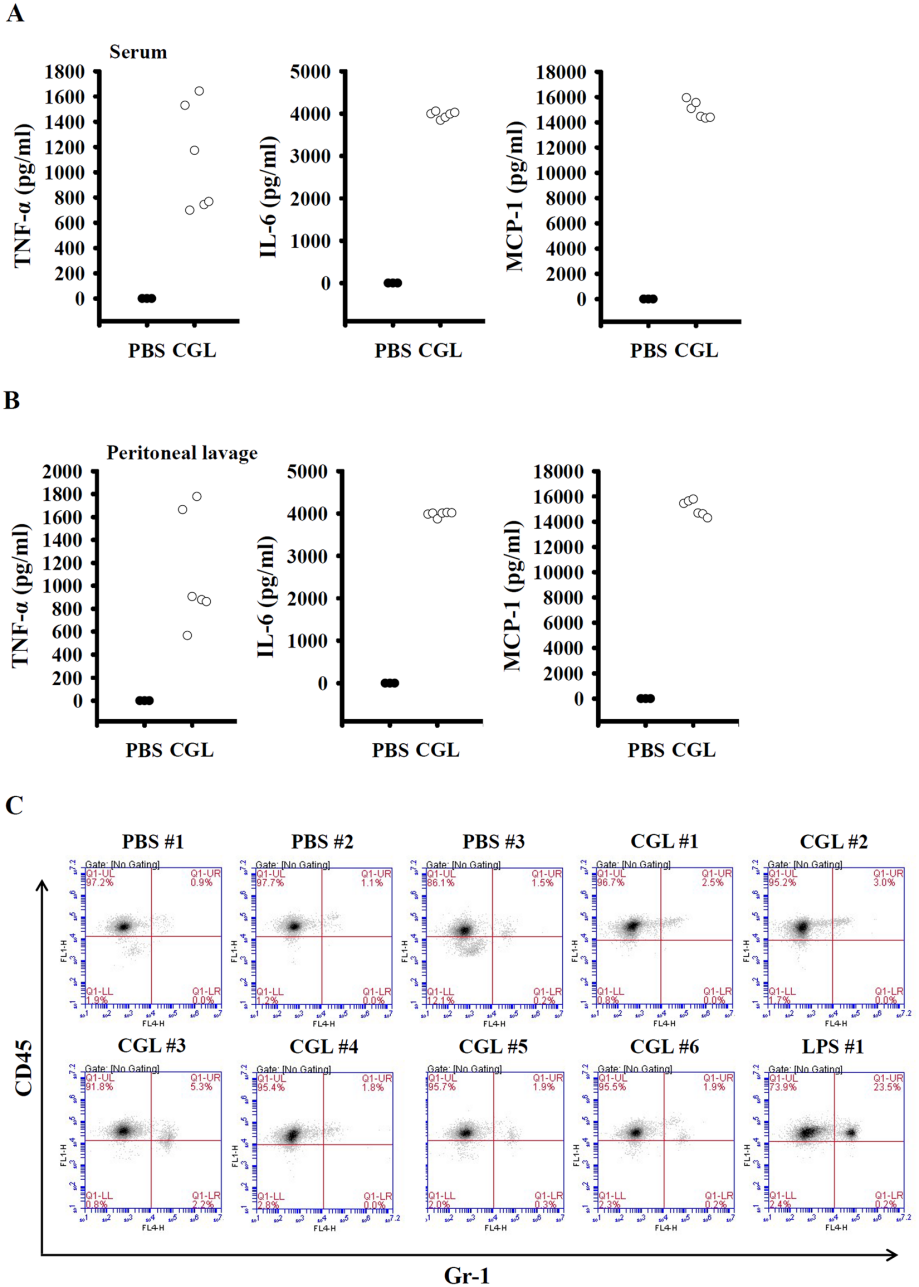
CGL induces cytokine expression *in vivo*. Mice were intraperitoneally injected with CGL (20 mg/kg), LPS (10 mg/kg) or PBS for 4 h. The levels of TNF-α, IL-6 and MCP-1 in serum (**A**) and peritoneal lavage (**B**) were measured by ELISA. The levels of peritoneal neutrophils infiltration were measured by flow cytometry (**C**).

To investigate whether CGL exhibited immune modulatory activities *in vivo*, we analysed the cytokine levels in the serum and peritoneal lavage of mice after intraperitoneal injections with CGL and PBS. We found that the levels of TNF-α, IL-6 and MCP-1 were elevated in the serum (Fig. 9A) and peritoneal lavage (Fig. 9B) of mice injected with CGL compared with those injected with PBS. Additionally, intraperitoneal injections with LPS caused significant neutrophil influx in the peritoneal cavities, i.e., one of the features of peritonitis. CGL injection did not induce neutrophil influx (Fig. 9C). These results indicated that CGL did not induce peritonitis *in vivo*. Furthermore, we did not observe any signs of acute pain in the mice after CGL injection, including vocalization, restlessness, porphyrin discharge and increased respiration.

## Discussion

A growing number of lectins are being isolated from marine organisms^8^. Marine lectins are structurally diverse, and their unique structures have a number of potential uses in biomedical applications. Although more than 300 species of marine lectins have been found, most of these investigations have focused on those with biomedical applications as antibacterial, antifungal, antiviral, antitumour, antinociceptive and anti-inflammatory agents^8, 29^. Lectins are characterized as immunomodulatory agents and can induce the production of certain cytokines and reactive species to induce efficient immune responses against tumours or microbial infections^30^. Unfortunately, there is limited data on the immunomodulatory activity of marine bivalve lectins compared with those of plant lectins. In this study, we found that the GalNAc/Gal -specific lectin, CGL, was able to activate macrophages by increasing their cytokine secretion and enhancing their bactericidal properties.

Just as lectins are diverse, they also have many different biological functions. For example, lectins from the green seaweed *Caulerpa cupressoides* var. lycopodium have anti-inflammatory activities, inhibiting cytokine production in carrageenan-induced rat-paw oedema models^31^. In contrast, lectins from the edible mushroom *Agrocybe aegerita* induce pro-inflammatory cytokine production by macrophages and promote the severity of caecal ligation and puncture-induced sepsis in mice^32^. In our previous studies, we demonstrated that CGL exhibited antibacterial and antifungal activities^18, 19^. Here, we further demonstrated that CGL not only induced cytokine expression but also increased the bactericidal activity of macrophages. We found that CGL induced ROS production (Fig. 3A), suggesting that the increased ROS may partially be responsible for the enhanced bactericidal activity since ROS generated from activated macrophages is an essential bactericidal component against intracellular bacteria^33^. Even though mitochondrial ROS generated from activated macrophages can contribute to macrophage bactericidal activity^34^, the effect of CGL on mitochondrial ROS production requires further investigation. Leukotrienes have also been demonstrated to enhance macrophage bactericidal activity against *Klebsiella pneumoniae* through the PKCδ-dependent activation of NADPH oxidase^35^. However, we did not collect data showing that CGL can induce leukotrienes in macrophages; rather, we showed that CGL activated PKCδ (Fig. 3C) and induced ROS production (Fig. 3A).

One of the interesting findings of this study was that CGL exhibited immune modulation properties. CGL induced cytokine production and also induced LPS tolerance. Even in the absence of LPS, CGL induced NO, TNF-α and IL-6 production, albeit at a relatively lower level than those induced by LPS. These results suggested that CGL was able to enhance immunity by activating macrophages and not causing severe inflammation, similar to LPS. Rather, CGL pre-treatment reduced pro-inflammatory mediator expression by LPS induction, suggesting that CGL ameliorated inflammation during acute infection. These results suggested that CGL could potentially be developed as an immune modulatory agent. It should be noted that CGL pre-treatment also resulted in IRAK2 degradation, a downstream signalling molecule of TLR4 (Fig. 7D). Despite this, CGL pre-treatment did not inhibit all LPS-mediated inflammatory responses, as it did not reduce TNF-α expression (Fig. 7A). In future studies, it would be interesting to determine how LPS may have induced TNF-α expression without IRAK2. One possible mechanism could be that LPS used two different signalling pathways in macrophages: MyD88/Mal/IRAKs and TRAM/TRIF/RIP1 for activating NF-κB and MAPKs and inducing NF-κB and MAPK-dependent cytokine production^36^.

LPS is a potent stimulator for macrophages and can induce cytokine production in macrophages even at low concentrations. Thus, when performing immune related studies, it is extremely important to test for possible LPS contamination in samples^37–39^. Although we carefully prepared CGL to avoid such contamination, we also used LPS-sequestering agent polymyxin B and the LAL test to further prevent LPS contamination. Additionally, we found that CGL induced cytokine production in macrophages through pathways different from LPS. For example, NO was induced significantly by LPS, but not by CGL (Fig. 2F). Additionally, in RAW264.7 macrophages, LPS induced TNF-α production through NF-κB^40^, but no change was observed in the TNF-α production in CGL-activated macrophages (Fig. 5C and E). Furthermore, LPS induced NF-κB activation through ROS^41, 42^, MAPKs^42^ and PI3-kinase^43^, but inhibitors of ROS (Fig. 6B), MAPKs (Fig. 6D) and PI3-kinase (Fig. 6E) did not significantly reduce CGL-mediated NF-κB activation. Finally, LPS pre-treatment induced tolerance to LPS by inhibiting TNF-α production, but CGL pre-treatment did not inhibit LPS-induced TNF-α production (Fig. 7A). These results, therefore, indicated that our CGL sample was LPS-free and induced cytokine expression in macrophages through signalling pathways different from those of LPS.

In this study, we demonstrated that CGL induced macrophage activation *in vitro* and promoted the expression of cytokines in a mouse model. Although CGL was derived from edible mussel and did not cause any significant toxicity in mice under these experimental conditions, more detailed toxicity tests (e.g., acute oral toxicity tests with a single high-dose administration of CGL and repeated dosages for 28 days) should be conducted before human use.

## Materials and Methods

### Materials

Natural CGL was isolated as described previously^16^. Recombinant CGL was prepared according to our previous study^19^ and further purified using Pierce High Capacity Endotoxin Removal Spin Columns (Thermo Fisher Scientific, USA). LPS (from *Escherichia coli* 0111:B4), N-acetylcysteine (NAC), PD98059, SP600125, SB203580, PDTC and mouse antibodies against mouse phospho-ERK1/2, phospho-JNK1/2, phospho-p38 and actin were purchased from Sigma-Aldrich (St. Louis, MO). Gö6976, Rottlerin, Wortmannin, LY294002, sc-3060, and antibodies against phospho-PKCα/δ, IL-1β, iNOS, COX-2 and IRAK2, as well as secondary antibodies were obtained from Santa Cruz Biotechnology (Santa Cruz, CA). IL-1β, IL-6, TNF-α and MCP-1 ELISA kits were purchased from R&D Systems (Minneapolis, MN). Pierce™ LAL Chromogenic Endotoxin Quantitation Kit was purchased from Thermo Scientific (Rockford, IL).

### Cell cultures

The murine macrophage cell lines RAW264.7 and J774A.1 and human THP-1 monocytes were obtained from American Type Culture Collection (Rockville, MD). RAW 264.7 macrophages stably expressing the gene for secreted embryonic alkaline phosphatase inducible by NF-κB (RAW-Blue™ cells) were purchased from InvivoGen (San Diego, CA). For the preparation of mice bone marrow-derived macrophages, marrow was collected from C57BL/6 mice femur and tibia and incubated for 7 days in culture medium containing M-CSF (Peprotech, London, UK). To induce monocyte-to-macrophage differentiation, the THP-1 cells were cultured for 48 h in RPMI-1640 medium supplemented with 100 nM phorbol 12-myristate 13-acetate (Sigma-Aldrich). Human PBMC were isolated from whole blood by Ficoll-Hypaque density gradient centrifugation method. Human MDM were obtained by culturing PBMC in 6-cm culture dishes for 7 days.

### Proteinase K and boiling treatment of CGL

For proteinase K treatment, 30 μg of CGL or 3 μg of LPS were incubated with or without 3 μg of proteinase K in a total reaction volume of 30 μl for 3 h at 50 °C. The samples were maintained on ice immediately after 50 °C incubation, and the samples were further analysed by SDS-PAGE and Coomassie Blue staining. For boiling treatment, 30 μg of CGL or 3 μg of LPS in a total reaction volume of 30 μl were incubated for 10 min at 100 °C or at 4 °C as a control. The samples were maintained on ice immediately after 100 °C incubation.

### Monosaccharide competition assay

Briefly, 100 μg of CGL were incubated with or without 1000 μg of GalNAc, GalN or Gal separately in total reaction volumes of 100 μl at 4 °C for 24 h. Cells were stimulated with CGL or monosaccharide-incubated CGL for 24 h, and TNF-α production was measured by ELISA.

### Detection of pro-inflammatory mediators and protein phosphorylation

The levels of cytokines and NO were measured by ELISA and Griess reaction, respectively. The expression levels of proIL-1β, COX-2, iNOS, IRAK2 and the phosphorylation levels of PKCα, PKCδ, ERK1/2, JNK1/2 and p38 in cells were measured by Western blot. Detailed procedures of the ELISA, Griess reaction and Western blot were described in our previous study^22^.

### Detection of ROS

Intracellular ROS production was measured by detecting the fluorescence intensity of 2′,7′-dichlorofluorescein, the oxidation product of 2′,7′-dichlorofluorescein diacetate (Molecular Probes, Eugene, OR). Briefly, RAW264.7 macrophages were incubated for 30 min with or without NAC (10 mM), for 30 min with H_2_DCFDA (2 μM), and then for 0–40 min with or without CGL (10 μg/ml). The fluorescence intensity of 2′,7′-dichlorofluorescein was detected at an excitation wavelength of 485 nm and an emission wavelength of 530 nm using a iMark™ Microplate Absorbance Reader (Bio-Rad Laboratories Inc., Hercules, CA, USA).

### NF-κB reporter assay

RAW-Blue™ cells were first incubated for 30 min with or without PDTC (30 mM), sc-3060 (0.1–3 μM) or NAC (10 mM) and then incubated for 24 h with or without CGL (1–10 μg/ml). In the LPS tolerance assay, RAW-Blue™ cells were incubated for 24 h with or without CGL (1–10 μg/ml) or LPS (0.1 μg/ml) and then changed to fresh medium and incubated for 24 h with or without LPS (1 μg/ml). The medium (20 μl) from the treated RAW-Blue™ cells was mixed with 200 μl of QUANTI-Blue™ medium (Invitrogen, Carlsbad, CA) in 96-well plates and incubated at 37 °C for 15 min. Secreted embryonic alkaline phosphatase activity was assessed by measuring the optical density at 655 nm using a microplate absorbance reader.

### Phagocytosis assay

RAW264.7 macrophages were infected with *E*. *coli* at 100 multiplicities of infection. After incubating for 1 h, the extracellular bacteria were removed by washing the cells three times with PBS. The cells were then incubated in medium containing 100 µg/ml of gentamicin to further eliminate the adherent extracellular bacteria. The number of viable bacteria within the cells was determined by counting the colony-forming units after 1 and 24 h postinfection.

### Quantitative real-time PCR analysis

To test for the PKCα and PKCδ expression in PKC shRNA-transfected or control shRNA-transfected cells, total mRNA from the cells was used to synthesize cDNA, followed by quantitative real-time PCR analysis with the QuantiTect SYBR^®^ Green RT-PCR Kit (Qiagen, Valencia, CA). The primer sequences used in this study were as follows: PKCα forward: 5′-CCCATTCCAGAAGGAGATGA-3′; PKCα reverse: 5′-TTCCTGTCAGCAAGCATCAC-3′; PKCδ forward: 5′-CAGACCAAGGACCACCTGTT-3′; PKCδ reverse: 5′-GCATAAAACGTAGCCCGGTA-3′; GAPDH forward: 5′-AAGGTCATCCCAGAGCTGAA-3′; and GAPDH reverse: 5′-CTGCTTCACCACCTTCTTGA-3′. The data for PKCα and PKCδ mRNA expression were determined from their optimized threshold values (C_T_ values) normalized against the C_T_ value of GAPDH as a percentage of the respective mRNA expression in the control shRNA-transfected cells.

### Ethical statement

Animal experiments were performed with the approval of the Institutional Animal Care and Use Committee of the National Ilan University (approval number: No. 102–40), according to the NIH Guide for the Care and Use of Laboratory Animals. The animal study was also approved by an ethical committee at the Ministry of Science and Technology of Taiwan (grant number: NSC 103-2923-B-197-001-MY3).

### Animal experiments

Experiments were performed on 8-week-old female C57BL/6 mice purchased from the National Laboratory Animal Breeding and Research Centre (Taipei, Taiwan). Briefly, 0.5 mg CGL was dissolved in 200 μl sterile PBS (CGL buffer), and 0.25 mg LPS was dissolved in 200 μl sterile PBS (LPS buffer). The mice were randomized into three groups: Group I: control, one intraperitoneal injection with sterile PBS (200 μl), n = 3; Group II: CGL treatment (20 mg/kg), one intraperitoneal injection with CGL buffer (200 μl on average, adjusted by body weight), n = 6; and Group III: LPS treatment (10 mg/kg), one intraperitoneal injection with LPS buffer (200 μl on average, adjusted by body weight), n = 1. Four hours after treatment, the serum and peritoneal lavage were collected for cytokine analysis. Infiltration by peritoneal neutrophils was analysed by flow cytometry after Gr-1 and CD45 staining.

### Statistical analysis

All values are mean ± SD. The data analysis involved one-way ANOVA with a subsequent Scheffé test.

## Electronic supplementary material

Supplementary information
