# Supplementary material for: A GalNAc/Gal-specific lectin from the sea mussel Crenomytilus grayanus modulates immune response in macrophages and in mice
Source: Sci Rep. 2017 Jul 24;7:6315. doi: 10.1038/s41598-017-06647-5 (PMC5524719; doi:10.1038/s41598-017-06647-5)
Supplement: Supplementary file 1 — Supplementary information [file 41598_2017_6647_MOESM1_ESM.pdf]

Supplementary information for

A GalNAc/Gal-specific lectin from the sea mussel *Crenomytilus grayanus* modulates immune response in macrophages and in mice

Oleg V. Chernikov[1,#], Wei-Ting Wong[2,3,#], Lan-Hui Li[4], Irina V. Chikalovets[1,5], Valentina I. Molchanova[1], Shih-Hsiung Wu[6], Jiahn-Haur Liao[6], Kuo-Feng Hua[2,7,*]

[1]G.B. Elyakov Pacific Institute of Bioorganic Chemistry FEB RAS, Vladivostok, Russia; [2]Department of Biotechnology and Animal Science, National Ilan University, Ilan, Taiwan; [3]Graduate Institute of Life Sciences, National Defense Medical Center, Taipei, Taiwan; [4]Department of Laboratory, Kunming Branch, Taipei City Hospital, Taipei, Taiwan; [5]School of Natural Sciences, Far Eastern Federal University, Vladivostok, Russia; [6]Institute of Biological Chemistry, Academia Sinica, Taipei, Taiwan; [7]Department of Pathology, Tri-Service General Hospital, National Defense Medical Center, Taipei, Taiwan

*Correspondence to:

Dr. Kuo-Feng Hua

Department of Biotechnology and Animal Science,

National Ilan University, Ilan, Taiwan

No. 1, Sec. 1, Shennong Rd., Ilan 260, Taiwan

Tel: +(886)-3-935-7626

Fax: +(886)-3-931-1526

E-mail: kuofenghua@gmail.com

[#]These authors contributed equally to this work.

Fig. S1

A (full gels of Fig.1A) **B (full gels of Fig. 1B)**

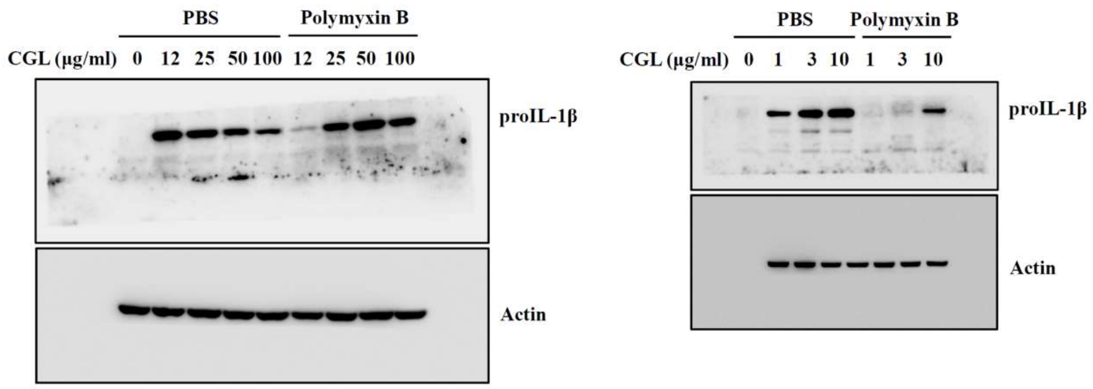

Fig. S1. The full-length gels of Figs.1A and 1B.

Fig. S2

A (full gels of Fig. 3C) **B (full gels of Fig. 3E)**

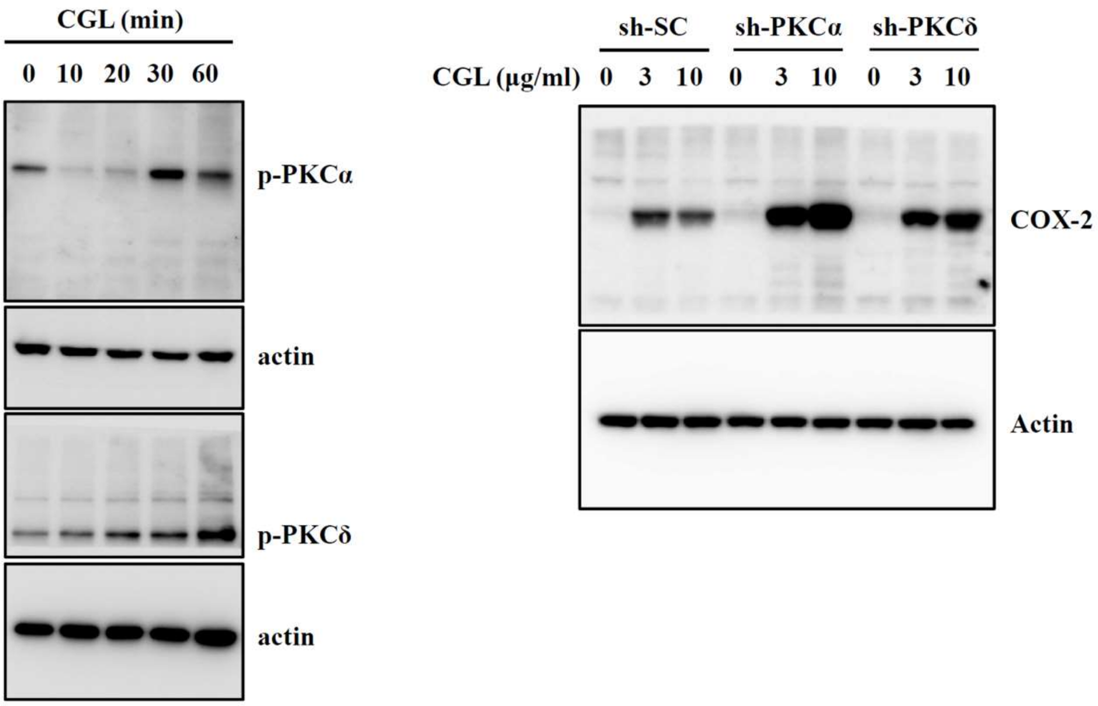

Fig. S2. The full-length gels of Figs. 3C and 3E.

Fig. S3

A (full gels of Fig. 4A)   B (full gels of Fig. 4B)

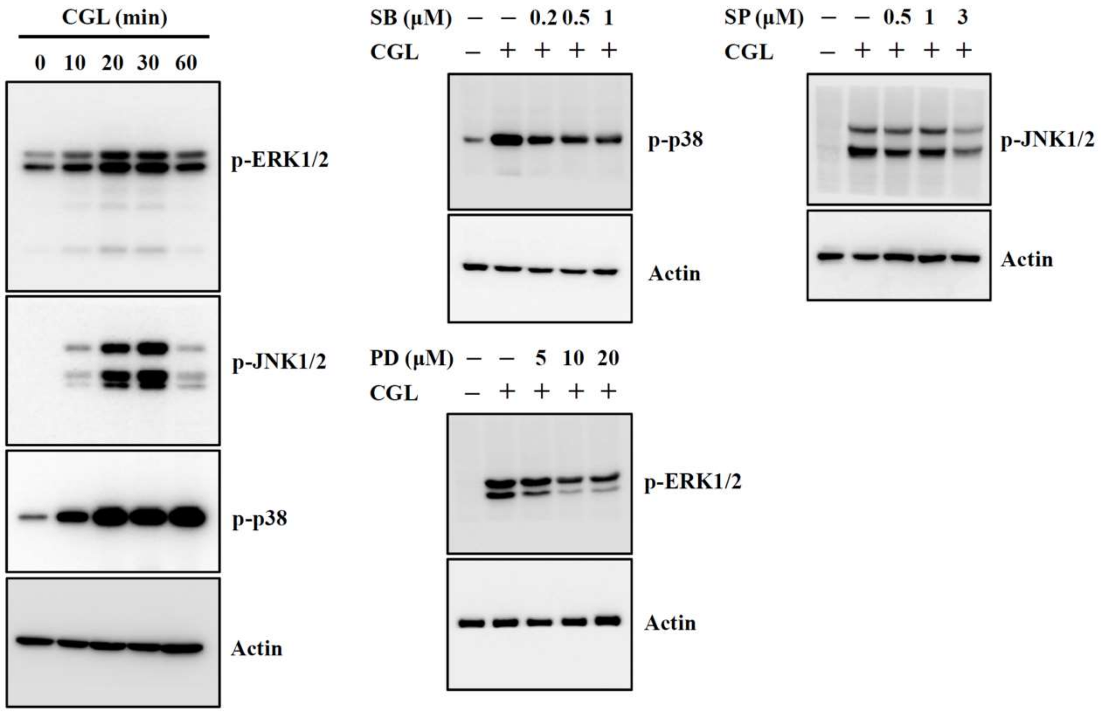

Fig. S3. The full-length gels of Figs. 4A and 4B.

Fig. S4 (full gels of Fig. 5B)

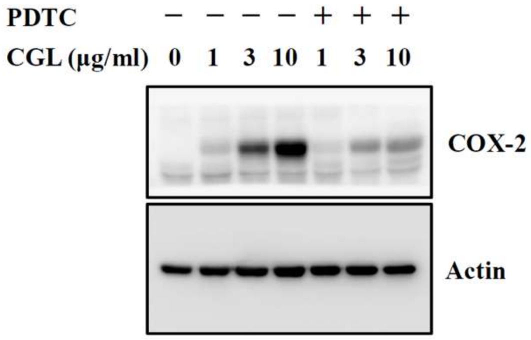

Fig. S4. The full-length gels of Fig. 5B.

Fig. S5 (full gels of Fig. 6A)

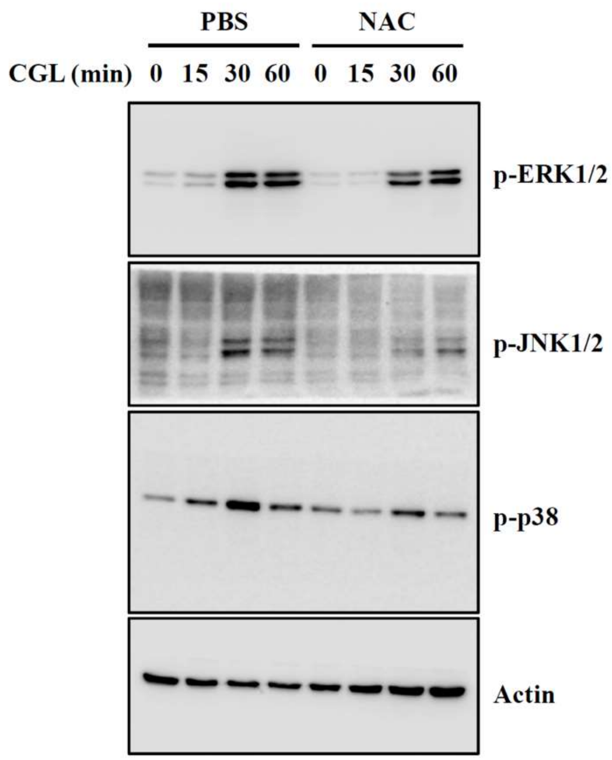

Fig. S5. The full-length gels of Fig. 6A.

Fig. S6

A (full gels of Fig. 7C)

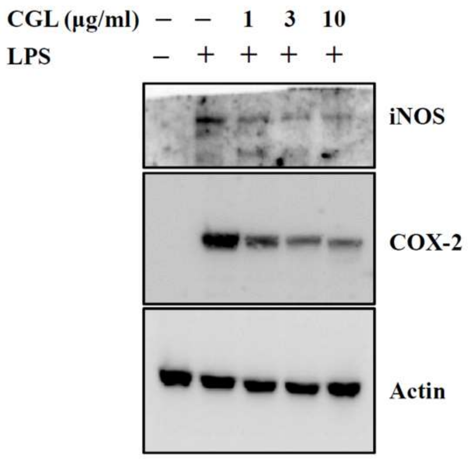

B (full gels of Fig. 7D)

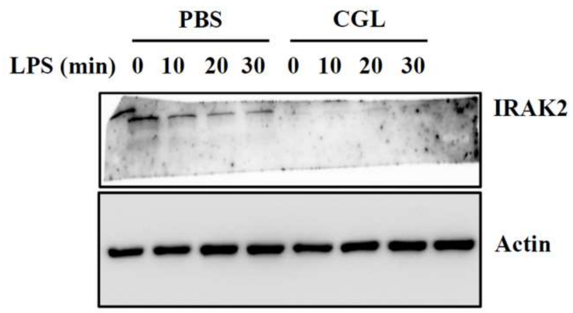

C (full gels of Fig. 7E)

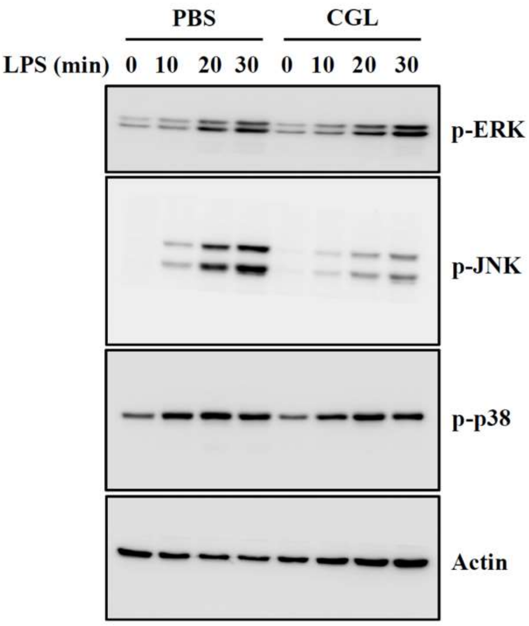

Fig. S6. The full-length gels of Figs. 7C, 7D and 7E.
